# Exploring perspectives and adherence to guidelines for adult spinal trauma in low and middle-income healthcare economies: A survey on barriers and possible solutions (part I)

**DOI:** 10.1016/j.bas.2022.100932

**Published:** 2022-08-19

**Authors:** Nicolò Marchesini, Andreas K. Demetriades, Oscar L. Alves, Francesco Sala, Andrés M. Rubiano

**Affiliations:** a Department of Neurosurgery, University Hospital Borgo Trento, Verona, Italy; b Department of Neurosurgery, Royal Infirmary, Edinburgh, UK; c Department of Neurosurgery, Leiden University, the Netherlands; d Department of Neurosurgery, Centro Hospitalar de Gaia/Espinho, Vila Nova de Gaia, Portugal; e Department of Neurosurgery, Hospital Lusiadas Porto, Porto, Portugal; f Neuroscience Institute, Universidad El Bosque, Bogotá, Colombia; g Meditech Foudation, Cali, Colombia

## Abstract

• Most spinal trauma occurs in low- and middle-income countries (LMICs), but some elements may limit the application of current guidelines.
• In LMICs, a respectable proportion of physicians treating spinal trauma is not aware of any guidelines on this topic.
• Most physicians managing spinal trauma in LMICs believe that following the guidelines may positively affect patient outcomes.
• Most believed they have the capability to apply, the guidelines, but variation according to income and geographical region exists.
• The perceived limitations and their relevance to guideline adherence vary across different income and geographic areas worldwide.
• Resource-targeted guidelines for spinal trauma are considered a valuable option to overcome the limitations of real-life application of the current guidelines.

## Abbreviations:

**CNS/AANS**: Congress of Neurological Surgeons/American Association of Neurological Surgeons
**DGOU**: Deutsche Gesellschaft für Orthopädie und Unfallchirurgie
**EA&P**: East Asia and Pacific
**E&CA**: Europe and Central Asia
**HICs**: high-income countries
**LA&C**: Latin America and the Caribbean
**LICs**: low-income countries
**LMICs**: low- and middle-income countries
**L-MICs**: lower-middle-income countries
**ME&NA**: Middle East and North Africa
**NICE**: National Institute for Health and Care Excellence
**SA**: South Asia
**SSA**: Sub-Saharan Africa
**TSI**: traumatic spinal injury
**U-MICs**: upper-middle income countries
**WFNS**: World Federation of Neurosurgical Societies

## 1 Introduction

It is estimated that around 13.8 million neurosurgical cases that require an operation arise each year worldwide and more than 80% of these occur in LMICs. Neurotraumas, including traumatic spinal injury (TSI), represent the commonest in everyday practice (Dewan et al., 2019). A relevant disparity in the distribution of cases between HICs and LMICs exists, with 101.477 new TSI occurring in the former and 843,316 in the latter (Kumar et al., 2018). Nevertheless, the distribution of the neurosurgical workforce around the globe is far from being homogeneous, with a neurosurgeon density estimated to range from zero (33 countries) to 58.95 (Japan) per 1,000,000 population. Thirty-five countries are estimated to have less than 1 neurosurgeon per 1,000,000, a number that falls significantly short of the desired ratio of 1 per 100,000 inhabitants. Almost invariably, the regions with the highest lack of neurosurgeons are represented by LMICs (Mukhopadhyay et al., 2019). This unequal distribution of the neurosurgical workforce, in addition to high disparities in the distribution of other health resources, technologies and infrastructures, could contribute to possible differences in the care offered to patients suffering from TSI (Calderón and Servén, 2014; WHO. World Health Statistics Organization, 2021).

The introduction and application of guidelines, recommendations and protocols could contribute to level, at least in theory, the differences existing among the various contexts. As they represent the best of evidence-based medicine, their application in all environments should be desirable. Indeed, helping clinicians in taking the best clinical decisions for neurotrauma patients, guidelines have been demonstrated to improve outcomes and reduce mortality (Sesperez et al., 2001; Curtis et al., 2011; Mullins and Mann, 1999; Celso et al., 2006). TSI does not represent an exception, as timely and appropriate treatment can prevent complications like limited mobility, postural deformities and chronic pain, which can cause considerable economic, social and medical consequences (Noonan et al., 2014; Ma et al., 2014).

In the last decades, significant progress has been done with the production of high-quality and comprehensive guidelines for the management of TSI. However, a not negligible proportion of the recommendations, implicitly or explicitly, require an efficient and readily available pre-hospital system, expensive diagnostic and surgical tools, and well-trained medical, surgical, anaesthesia and post-operative care staff (Yue et al., 2016; Maschmann et al., 2019; Yue et al., 2017; Sharif et al., 2020; Sánchez et al., 2020; Alves et al., 2020; Peev et al., 2020; Zileli et al., 2020a; ParthibanMehmet Zileli, 2020; Zileli et al., 2020b; Takami et al., 2020). A partial or total absence of the above-mentioned components, could limit the application of the recommendations themselves and, possibly, affect the quality of the care offered and the outcomes for the patients.

The present survey aims to map the adherence to some of the recommendations for the management of TSI in LMICs, trying to identify possible barriers and serving as guidance for desirable future solutions.

The high volume of data obtained and their richness made it valuable for the reader to divide the results and the discussion into three manuscripts, each presenting a different but interrelated topic.

In the current paper, we analyze the demographics and characteristics of the respondents and we focus on their perspectives on the current guidelines. Other aspects specifically related to prehospital care and in-hospital heterogeneity will be analyzed and discussed in a subsequent series of articles (“*A survey on early management of spinal traumas in low- and middle-income countries: from the scene of injury to diagnosis (Part II),”* where we will analyze data about pre-hospital care and diagnostics for TSI in LMIC and “*Secondary damage management of acute traumatic spinal cord injury in low- and middle-income countries: a survey on a global scale (Part III)”* where we will explore some aspects about the treatment of spinal injured patients (medical, surgical and rehabilitation)).

## 2 Methods

An electronic online survey was designed and uploaded into Google Modules (Google©) and Wenjuan©. Thirty-four questions (Appendix I) were logically structured into six main sections. Section one investigated demographic and general information of the respondent and the Institution of employment. Sections two to five were formulated aiming to explore the capacity of adherence to specific WFNS recommendations across different phases of care (pre-hospital and emergency, diagnostics, treatment and rehabilitation). The last section explored some opinions of the respondents regarding guidelines and recommendations. The topics were voluntarily reduced to a limited number, balancing acceptable completeness and conciseness.

Likert scales were used to assess the rate of agreement with specific sentences. Barriers to the application of the guidelines were asked to be graded from 0 (no important at all) to 3 (very important).

The questionnaire was targeted at physicians treating TSI in LMICs. Countries were classified by income (LICs=low-income, L-MICs=lower-middle-income, U-MICs=upper-middle income) and region (EA&P=East Asia and Pacific, E&CA=Europe and Central Asia, LA&C=Latin America and the Caribbean, ME&NA=Middle East and North Africa, SA=South Asia and SSA=Sub-Saharan Africa) according to the last Word Bank Classification. (World Bank) Between the 20^th^ of May and the August 20, 2021 the survey was disseminated online by social media (Facebook, Whatsapp, Telegram, Instagram, Twitter), emails and webinars. Data were prospectively collected and the results were tabulated in a Microsoft Excel spreadsheet. Calculation of a response rate was not possible because of the wide distribution of the questionnaire through social media. The order in which results are presented does not necessarily follow the order of the questions in the questionnaire. Not all questions were included in the final analysis. Pearson's chi-square test was used to assess measures of association in frequency tables. For statistical significance values of p<0.05 were considered. Statistical analysis was performed by commercially available software (Microsoft Excel).

## 3 Results

### 3.1 Demographics

**Fig. 1:**
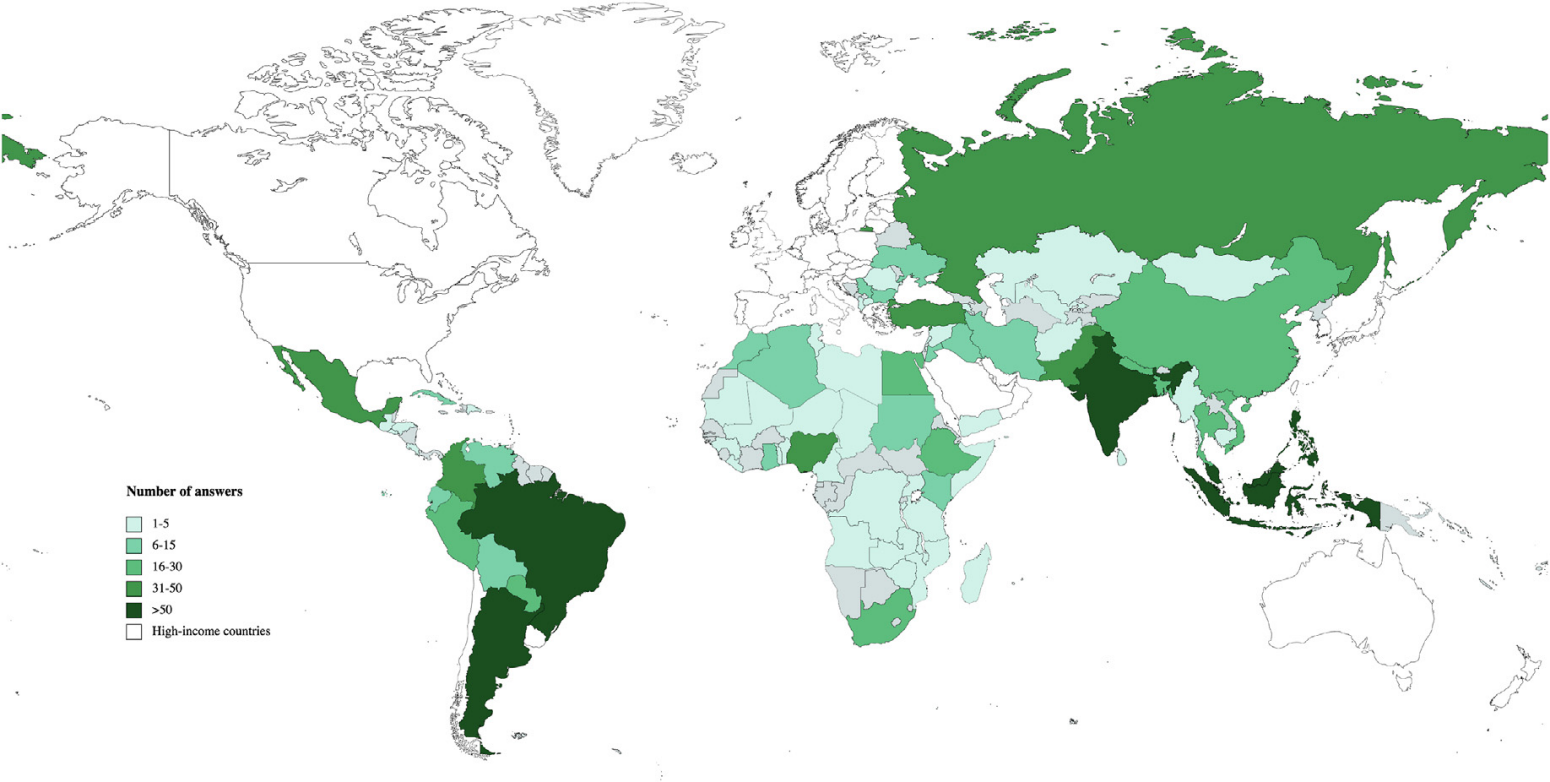
World map showing the global distribution of the answers received to the survey. Grey areas indicate LMICs from which no answers were obtained and white areas HICs (not included in the survey).

One-thousands-one hundred fifty-four answers (1154) were obtained from 79 LMICs. Most answers came from L-MICs and U-MICs (1103; 95.6%). The regions with the highest rate of responses were EA&P and LA&C (597; 51.7%) and the country with the highest number of answers was India (135). The mean number of answers was 17, the median was 4. Detailed geographical distribution is depicted in Fig. 1. Most answers came from males (1041; 90.2%) and the geographical region with the highest female representation was EA&P (17.8%). The most represented age group was 30–49 years (828; 71.8%) and the income region with the youngest representation was LICs (10; 19.6%). Most answers were obtained from Specialists in Neurosurgery (564; 48.9%) and Specialists in Orthopedics (361; 31.3%). The geographical area with the highest proportion of Specialists in Neurosurgery was E&CA (70/98; 71.4%) while the highest rate of Specialists in Orthopaedics was in EA&P (105/297; 35.4%) and SA (79/223; 35.4%). Overall, 39.3% (454/1154) stated to be experienced in managing TSI for longer than 10 years. Altogether, 51.5% (594) stated to practice in Institutions with a medium level of resources. In LICs, a higher proportion was found to practice in low-resources Institutions (21/52; 41.2%), compared to L-MICs (52/558; 9.3%) and U-MICs (54/545; 9.9%). Overall, 39.3% (454) stated that their Institution served a population from 1 to 5 million. In LICS, 60.8% (31/51) declared to treat more than 5 millions people when compared to L-MICs (181/558; 32.4%) and U-MICs (107/545; 19.6%). The vast majority (98.7%; 1039/1154) answered to treat (regularly or occasionally) patients with spinal cord injury. Demographic data are summarized in Table 1.

**Table 1:** Demographics of the 1154 respondents to the questionnaire (LICs=low-income countries, L-MICs=lower-middle-income countries, U-MICs=upper-middle-income countries, EA&P=East Asia and Pacific, E&CA=Europe and Central Asia, LA&C=Latin America and Caribbean, ME&NA=Middle East and North Africa, SA=South Asia, SSA=Sub-Saharan Africa).

Demographic	Total(%)	LICs	L-MICs	U-MICs	EA&P	E&CA	LA&C	ME&NA	SA	SSA
**Total (%)**	1154(100)	51 (4.4)	558(48.4)	545(47.2)	297(25.7)	98(8.5)	300(26)	108(9.4)	223(19.3)	128(11.1)
**Sex**										
Male	1041(90.2)	47 (92.2)	498(89.2)	496(91)	244(82.2)	89(90.8)	279(93)	98(90.7)	214(96)	117(91.4)
Female	113(9.8)	4(7.8)	60(10.8)	49(9)	53(17.8)	9(9.2)	21(7)	10(9.3)	9(4)	11(8.6)
**Age (years)**										
<25	3(0.3)	0(0)	0(0)	3(0.6)	0(0)	2(2)	1(0.3)	0(0)	0(0)	0(0)
25-29	67(5.8)	10(19.6)	39(7)	18(3.3)	20(6.7)	9(9.2)	4(1.3)	5(4.6)	20(9)	9(7)
30-49	828(71.8)	37(72.5)	416(74.6)	375(68.8)	227(76.4)	70(71.4)	190(63.3)	69(63.9)	167(74.9)	105(82)
50-69	246(21.3)	4(7.8)	99(17.7)	143(26.2)	48(16.2)	16(16.3)	100(33.3)	32(29.6)	36(16.1)	14(10.9)
≥70	10(0.9)	0(0)	4(0.7)	6(1.1)	2(0.7)	1(1)	5(1.7)	2(1.9)	0(0)	0(0)
**Current job title**										
Consultant in Neurosurgery	564(48.9)	24(47.1)	249(44.6)	291(53.4)	122(41.1)	70(71.4)	179(59.7)	50(46.3)	95(42.6)	48(37.5)
Consultant in Orthopedics	361(31.3)	7(13.7)	174(31.2)	180(33)	105(35.4)	14(14.3)	94(31.3)	34(31.5)	79(35.4)	35(27.3)
Neurosurgery trainee	130(11.3)	14(27.5)	77(13.8)	39(7.2)	49(16.5)	10(10.2)	9(3)	19(17.6)	18(8.1)	25(19.5)
Orthopedic trainee	37(3.2)	1(2)	25(4.5)	11(2)	6(2)	1(1)	6(2)	2(1.9)	13(5.8)	9(7)
Other	62(5.4)	5(9.8)	33(5.9)	24(4.4)	15(5.1)	3(3.1)	12(4)	3(2.8)	18(8.1)	11(8.6)
**Experience with spinal trauma (years)**										
<5	393(34.1)	31(60.8)	216(38.7)	146(26.8)	111(37.4)	24(24.5)	76(25.3)	31(28.7)	85(38.1)	66(51.6)
5-10	307 (26.6)	15(29.4)	151(27.1)	141(25.9)	83(27.9)	24(24.5)	67(22.3)	30(27.8)	63(28.3)	40(31.3)
>10	454(39.3)	5(9.8)	191(34.2)	258(47.3)	103(34.7)	50(51)	157(52.3)	47(43.5)	75(33.6)	22(17.2)
**Level of resources of the Institution**										
Low level	127(11)	21(41.2)	52(9.3)	54(9.9)	31(10.4)	10(10.2)	31(10.3)	8(7.4)	18(8.1)	29(22.7)
Medium level	594(51.5)	23(45.1)	325(58.2)	246(45.1)	167(56.2)	47(48)	127(42.3)	63(58.3)	121(54.3)	69(53.9)
High level	433(37.5)	7(13.7)	181(32.4)	245(45)	99(33.3)	41 (41.8)	142(47.3)	37(34.3)	84(37.7)	30(23.4)
**Population served**										
<1 million	381(33)	7(13.7)	146(26.2)	228(41.8)	96(32.3)	30(30.6)	140(46.7)	36(33.3)	56(25.1)	23(18)
1–5 million	454(39.3)	13(21.5)	231(41.4)	210(38.5)	130(43.8)	38(38.8)	123(41)	40(37)	87(39)	36(28.1)
>5 million	319(27.6)	31(60.8)	181(32.4)	107(19.6)	71(23.9)	30(30.6)	37(12.3)	32(29.6)	80(35.9)	69(53.9)
**Spinal cord injury cases treatment**										
Yes, regularly	764(66.2)	37(72.5)	405(72.6)	322(59.1)	164(55.2)	59(60.2)	183(61)	74(68.5)	192(86.1)	92(71.9)
Yes, occasionally	375(32.5)	14(27.5)	149(26.7)	212(38.9)	131(44.1)	35(35.7)	114(38)	32(29.6)	28(12.6)	35(27.3)
No, never	15(1.3)	0(0)	4(0.7)	11(2)	2(0.7)	4(4.1)	3(1)	2(1.9)	3(1.3)	1(0.8)

### 3.2 Guidelines awareness

**Fig. 2:**
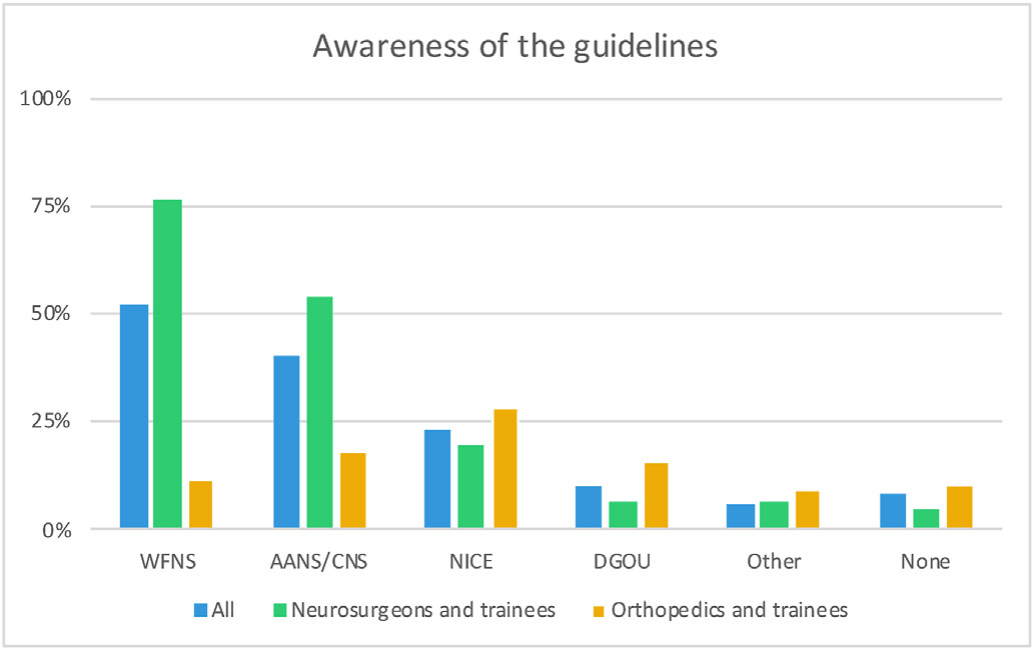
Bar chart showing the rate of awareness of some of the existing guidelines for spinal trauma by the whole sample (1154), specialists and trainees in neurosurgery (694) and specialists and trainees in orthopedics (398). The rate of awareness for the WFNS and AANS/CNS guidelines was significantly higher for neurosurgeons (76.2% vs 10.8%; p<0.0001) and (53.7% vs 18.1%; p<0.0001) while the rate of awareness of the DGOU guidelines was higher for orthopedics (15.8% vs 6.2%; p<0.0001).

Overall, 8.2% (95/1154) stated not being aware of any guidelines for the management of TSI. LICs respondents reported the highest rate of non-awareness (10/51; 19.6%). Amongst the listed guidelines (WFNS, CNS/AANS (Congress of Neurological Surgeons/American Association of Neurological Surgeons), NICE (National Institute for Health and Care Excellence), DGOU (Deutsche Gesellschaft für Orthopädie und Unfallchirurgie)), the WFNS guidelines were the best known (598; 51.8%). This was true both for the whole sample and after stratification both according to the geographical and income area. The region with the highest awareness of the WFNS guidelines was EA&P (67/98; 68.4%) and the one with the least was LA&C (128; 42.7%). There were significant differences in the rate of awareness of the different guidelines between neurosurgeons/trainees in neurosurgery vs orthopaedics/trainees in orthopaedics, with the WFNS and AANS/CNS guidelines best known by neurosurgeons (p<0.0001) and DGOU by orthopaedics (p<0.0001) (see Fig. 2).

### 3.3 Trust in guidelines' capability to affect outcomes

Overall, the vast majority declared to believe (agree or strongly agree) that following the guidelines for the management of TSI may positively affect patients’ outcomes (1095; 94.9%) while the rate of disagreement (disagree or strongly disagree) ranged from 0 to 1.3%. Even though the general rate of agreement was high, there were differences in the distributions of “strongly agree” and “agree” answers, both amongst income and geographical areas. The income area with the highest rate of “strongly agree” was LICs (38; 74.5%), followed by L-MICs (334; 60.4%) and U-MICs (333; 61.1%). The geographical area with the highest rate of “strongly agree” was SSA (100; 78.1%), followed by E&CA (65; 66.3%), LA&C (194; 64.7%), ME&NA (64; 59.3%), SA (128; 57.4%) and EA&P (157; 52.9%). No significant differences were found between neurosurgeons and orthopaedics.

### 3.4 Self-perception of the possibility to apply the guidelines

**Fig. 3:**
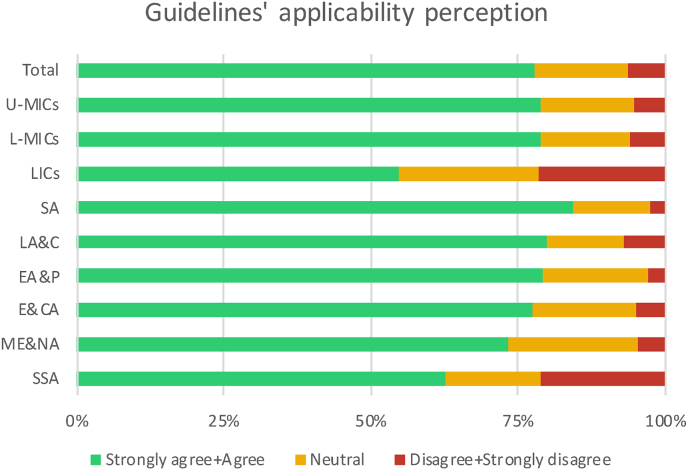
Bar chart showing the differences in the perceived capability to apply the guidelines for the management for spinal trauma. Results are stratified according to economic (LICs, L-MICs and U-MICs) and geographic area (SA; LA&C, EA&P, E&CA, ME&NA, SSA).

Overall, the majority stated (agree or strongly agree) to have the perception to have the capability to apply, in their environment, the guidelines for the management of TSI (897; 77,7%). However, differences were found when stratifying the answers according to the economic and geographic area (Fig. 3). The economic area with the lowest rate of agreement was LICs (28; 54.9%) and the geographic area was SSA (80; 62.5%).

### 3.5 Perceived limits in the application of the guidelines

**Fig. 4:**
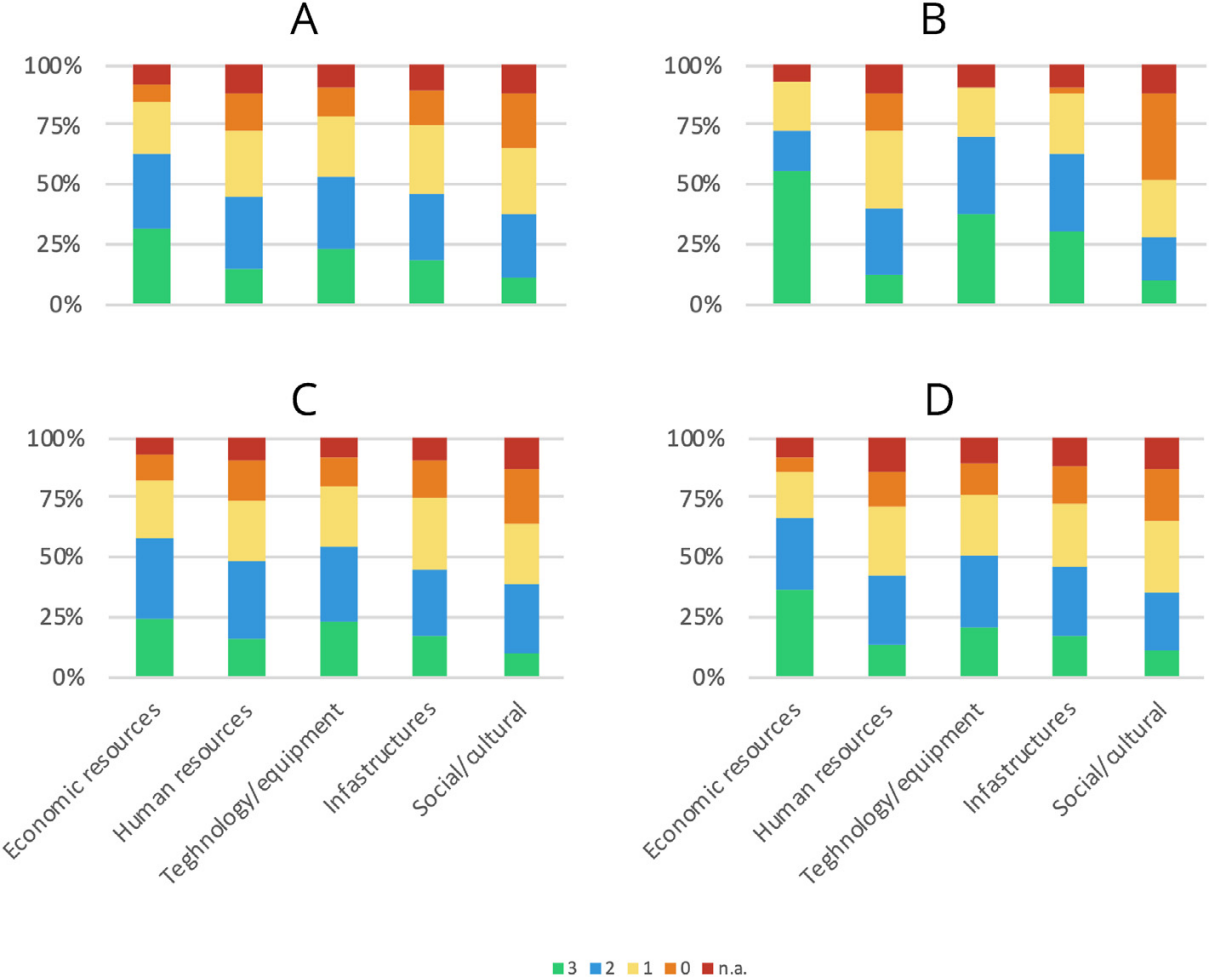
Relevance of some possible limits in the application of the guidelines for the management of spinal trauma. Results from the whole sample (A) and results stratified according to the resources (B=LICs, C=L-MICs, D=U-MICs). n.a.= not answering.

Perceived limits in the application of the guidelines for the management of TSI were explored amongst those who had not answered “strongly agree” to the question referring to the previous paragraph (737/1154; 63.9%). Overall, the most important (rate 2+3) limits were the lack of economic resources (63%) and lack of technology and equipment (53.5%) while social and cultural reasons were the least important (37.1%). Lack of economic resources was the most important limit in all the income regions. However, in LICs the rate of “3” was higher than in L-MICs and U-MICs (55% vs 35.9% vs 24.6%) (see Fig. 4). Lack of economic resources was also found as the main limitation (rate 2+3) in every geographic area, with differences according to the region: 70.4% SSA, 70% EA&P, 60.3% LA&C, 60.2% SA, 58.4% E&CA and 48.4% ME&NA. The region with the highest perception of lack of human resources as a limit was EA&P (53.55%), while for lack of technology/equipment and infrastructures it was LA&C 58.7% and 58.5% respectively. Social/cultural reasons were important limits (rate 2+3) for 48.5% of EA&P respondents.

### 3.6 Stratified guidelines

**Fig. 5:**
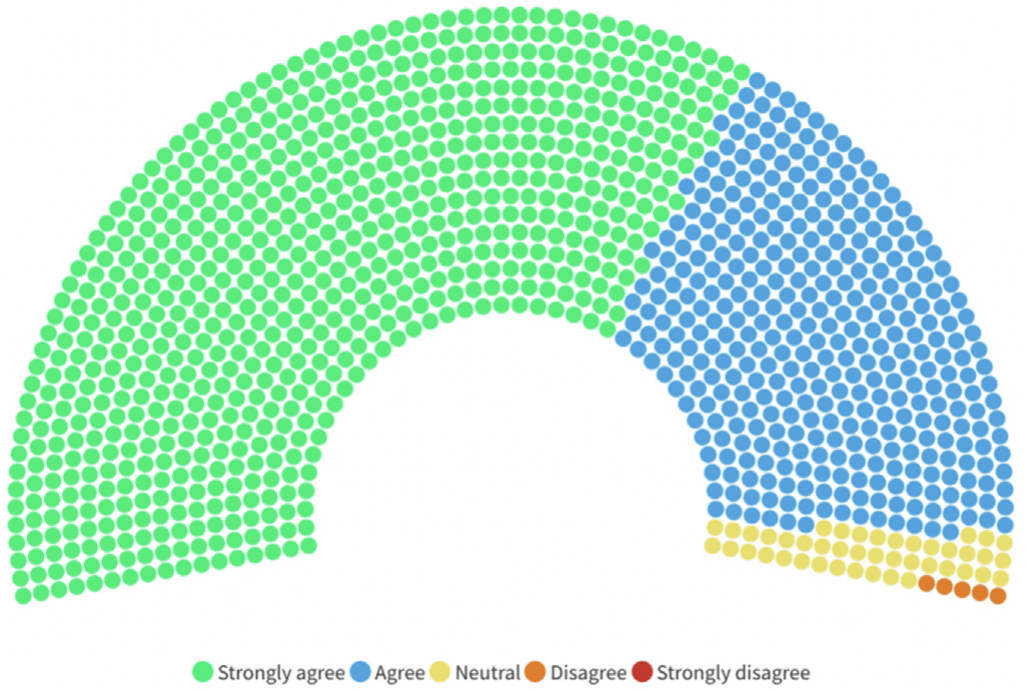
Rate of agreement of the whole sample (1154) with a sentence asking whether the introduction of stratified guidelines would be considered helpful to better treat spinal trauma patients and possibly improve patients' outcomes. Each dot corresponds to a respondent. No “strongly disagree” answers were obtained.

The vast majority (1106; 95.8%) agreed (366; 31.7%) or strongly agreed (740; 64.1%) with a sentence exploring whether or not stratified guidelines for the management of TSI could be useful to better treat patients and, possibly, improve their outcomes while only five disagreed (four from LA&C and one form SA) and none strongly disagreed (see Fig. 5).

The proportion of “strongly agree” was higher in LICs (38; 74.5%) and in SSA (98; 76.6%) and lower in U-MICs (338; 62%) and in EA&P (178; 59.9%).

No significant differences were found between neurosurgeons and orthopaedics.

## 4 Discussion

According to the latest World Bank population data-set, more than 6.5 of the 7.7 billion global population currently live in LMICs (The World Bank Population Total, 2021). As a whole, LMICs include 138 different regions across the globe, and those sampled in the present study correspond to 57.7% of the total amount. Although this percentage may look only moderately significant, in these countries live more than 6.2 billion people, which corresponds to 96.1% of the LMICs and 81% of the global population (Kumar et al., 2018).

Considering the differences (economic, social, political) amongst the included income and geographic areas, it's not difficult to hypothesize that the care that is being offered across the globe to TSI patients could be, at least, heterogeneous. These inequities may become even more evident if in the analysis we'd consider HICs, where the gross net income per capita per year is at least more than ten times higher than in LICs. (World Bank).

For these reasons, differences in outcomes for TSI across contexts with different resources might be significant, even if such data are lacking for many regions (Chhabra et al., 2018). The analysis of patients' outcomes and specific factors that might affect them goes beyond the scope of the present study and future research might help to unshadow which aspect/s is more detrimental (resources, lack of guidelines' awareness/knowledge …). However, some of our findings may help raise awareness of some key variabilities in the management of this condition that might influence outcomes themselves. Different levels of economic and human resources, infrastructures, lack of materials and equipment, unawareness and social limitations are only a few of the many factors that may affect together the adherence to guidelines. In turn, the lack of adherence to guidelines might be responsible for heterogeneous patients’ care.

The efforts to make homogeneous across the globe the management of TSI started more than two decades ago, resulting in the publication in 2002 in *Neurosurgery* of the first evidence-based guidelines for the management of cervical spine and spinal cord injury (Hadley, 2002a). The Section on Disorders of the Spine and Peripheral Nerves of the American Association of Neurological Surgeons and the Congress of Neurological Surgeons identified the “best care” strategies for all aspects of care, including the pre-hospital phase, neurological and radiographic assessment, medical management, closed reduction and specific treatment options, both operative and nonoperative. A strict methodology was followed, aiming to eliminate flaws that could arise from expert opinion and clinical experience (Hadley, 2002b). From that initial experience, recommendations were updated and other neurosurgical, orthopaedics and anesthesiology societies introduced other guidelines designed by different methodologies (Walters et al., 2013; Fehlings et al., 2017; Spinal, 2016; Roquilly et al., 2020; Hoffmann et al., 2018). The WFNS guidelines for cervical and thoracolumbar injuries are the most recent of this series (Zileli et al., 2020a; Sharif and Zileli, 2021). It is worth noting that the existing guidelines, in general, don't take explicitly into account the possibility that some of their recommendations may require components that are inadequate/absent/lacking in some regions of the world. Furthermore, the distribution of the guidelines may rely on channels that may be unable to reach all the final recipients, with an indirect impact on patients' management. This is confirmed, for example, by the fact that nearly one out of five LICs respondents seems not to be aware of any guideline and that the WFNS guidelines, even though published open-access, reached only around half of the spinal surgeons in our sample. Indeed, specialists in one field tended to have a higher awareness of the guidelines issued by societies of their speciality (both specialists and trainees). On one side, this is a common consequence of the ultra-specialization of modern medicine (Ekelund et al., 2013). On the opposite, as TSI may be considered a multidisciplinary issue, a high level of interplay and knowledge sharing may be desirable between neurosurgeons, orthopaedics, anesthesiologists and other providers (Alizo et al., 2018). An interesting aspect to explore for future research, but that wasn't included in our analysis, would be the awareness of the guidelines by non-medical health providers (i.e. pre-hospital care providers or physiotherapists).

Surveys of other surgical specialities explored awareness, knowledge, confidence and adherence to the respective guidelines (Cooney et al., 2004; Singh and Mayahi, 2004; Aarts et al., 2012; Buscaglia et al., 2009). A recent survey about the adherence to severe traumatic brain injury guidelines concluded that they may be hardly followed in LMICs, due to different pre-hospital, intensive and surgical care conditions that influence clinical and surgical behaviour (Saraceno et al., 2021). However, these topics for TSI guidelines hadn't been explored so far and data for comparisons with analogous studies are missing.

The level of confidence in guidelines' capability to positively affect patients’ outcomes was significant for the vast majority of our sample. However, it may be meaningful that the level of confidence was higher in low-resource settings and geographical areas with a high number of LICs, like SSA. This could ultimately outline the important role that guidelines may play in contexts where resources are scarcer.

Although the majority of our sample did feel to be able to apply the guidelines in daily clinical practice, around one out of four did not. It's relevant that this last proportion was higher in LICs, reaching almost 50%, while in middle-income regions this value nearly reached 80%.

Several barriers have been suggested as limits for the application of clinical practice guidelines in other specialities. However, it may be difficult to generalize the improvement to guideline adherence as barriers may be different from setting to setting (Cabana et al., 1999). In our sample, the lack of economic resources was universally indicated as the most important limiting factor for the application of the guidelines, independently from the economic or geographic area, even if its relevance resulted increasingly higher in lower resources areas. This result was somehow expected: as guidelines mainly focus on the early management of spinal injuries and as a significant proportion of costs derive from this phase, a lack of economic resources can explain such difficulties in applying the recommendations (Cao and Krause, 2020). The lack of materials/equipment can be a direct consequence of a lack of economic resources. However, this may be also explained by the progressive privatization of health systems in some low-resource regions, with a requirement of authorizations from private companies for operations and implants, that have generally not improved access to health services for some vulnerable groups (Waitzkin et al., 2007; Luzuriaga and Bahia, 2017; Guiroy et al., 2021).

To address the limit of applicability of the purely evidence-based guidelines in some LMICs environments, some pioneering studies on traumatic brain injury proposed protocols of treatment that span different levels of resources and complexity. Such guidelines were developed by a mixed methodology, joining evidence review and consensus of experts to fill gaps in knowledge (Rubiano et al., 2020). The respondents to our survey expressed a highly positive outlook towards similar guidelines for the management of TSI if they were available. This would follow recent beliefs that it may be desirable that the guidelines take into consideration region-specific factors, from a global health perspective (Saraceno et al., 2021).

## 5 Limitations

To the best of our knowledge, this survey is the first study exploring the adherence to TSI management guidelines in settings with limited resources. The survey was disseminated by social media platforms, emails, and presentations at webinars and for this reason, it was not possible to calculate the response rate. The survey was only in English and no-English speakers could have chosen not to answer. A possible bias common to all surveys with optional participation is that the respondents may have a higher interest in the examined topic when compared to non-responders. Although our sample included a considerable number of respondents from a large number of LMICs, the results may be not exactly representative of all LMICs realities and scenarios. The likelihood of clustering results with multiple respondents from the same institution is real. Moreover, even if our sample examined both neurosurgeons' and orthopaedics’ perspectives, in some regions of the world spinal traumas may be managed by only one or both specialities. However, a quantitative analysis goes beyond the scope of the study.

## 6 Conclusions

Most physicians treating TSI in LMICs seem to be aware of at least one guideline for the management of this condition, with some region and speciality-specific differences. The vast majority stated to be confident that the application of guidelines for the management of TSI could be helpful to positively affect patients’ outcomes, with a higher rate of agreement in low-resources settings. A moderately high proportion seemed to perceive to be able to apply the guidelines in their daily clinical practice, although with differences according to economic and geographic area. The lack of economic resources was pointed out as the main barrier to the application of the guidelines in all economic and geographic groups, although with different relevance according to the region. From a global health perspective, the vast majority agreed that resource-targeted guidelines may be helpful to better treat TSI patients and possibly improve their outcomes.

## Declaration of competing interest

The authors declare that they have no known competing financial interests or personal relationships that could have appeared to influence the work reported in this paper.
