# Supplementary material for: Exploring perspectives and adherence to guidelines for adult spinal trauma in low and middle-income healthcare economies: A survey on barriers and possible solutions (part I)
Source: Brain Spine. 2022 Aug 19;2:100932. doi: 10.1016/j.bas.2022.100932 (PMC9560659; doi:10.1016/j.bas.2022.100932)
Supplement: Multimedia component 1 [file mmc1.docx]

**Recommendations for the management of spinal trauma in low-middle income countries (LMICs): a survey on barriers and possible solutions.**

*It is estimated that more than 80% of the global cases of spinal trauma arise in regions of the world with limited economical, human, technological and infrastructural resources. The application of protocols, guidelines and recommendations could enhance the care offered to spinal trauma patients, improving outcomes and reducing mortality. Nevertheless, the resource limitations could constitute an essential barrier to their widespread and use.*

*This survey is addressed to physicians managing spinal trauma in LMICs. The aim of the study is to map the extent of adhesion to the most recent recommendations for spinal trauma among physicians in LMICs, trying to identify barriers and seek possible solutions to overwhelm this limit.*

**Questions:**

1. Select your age group

- <25
- 25-29
- 30-49
- 50-69
- 70 or more

1. Select your gender

- Male
- Female

1. Where are you currently practicing? (specify the country)
2. In which country did you complete your residency or where are you currently enrolled in a residency program?
3. What is your actual practice status?

- Resident in Neurosurgery
- Resident in Orthopedics
- Specialist in Neurosurgery
- Specialist in Orthopedics
- Other

1. Select your time of experience in treating spinal trauma

- <5 years
- 5-10 years
- >10 years

1. Select the status of the main Institution where you treat spinal trauma (more than one option available)

- Private
- Public
- Mixed
- Academic
- Non Academic

1. Select the level of resources at this main facility

- Low level of resources (non-specialized care)
- Medium level of resources (specialized care)
- High-level of resources (sub-specialized care)

1. Select the population who beneficiate from care in this main facility (being or not a referral centre

- <1 million
- 1-5 millions
- >5 millions

1. In this Institution, do you treat patients with spinal trauma and associated neurological deficit?

- Yes, regularly
- Yes, occasionally
- No, never

1. In your region, the pre-hospital care of patients with suspected spinal cord injury is performed by:

- Basic providers (lay person/first responder)
- Advanced providers (paramedic/nurse/physician)
- There is no formal pre-hospital response (transported by private/public vehicles)

1. Select the available options of transportations of patients with spinal trauma in your region (more than one option available)

- Air
- Ground
- Water
- All
- None

1. In your facility, patients at high risk of spinal cord injury arrive immobilized with a hard cervical collar from the scene?

- Yes, regularly
- Yes, occasionally
- No, never,
- I don't receive patients at high risk of spinal cord injury

1. In your facility, patients at high risk of spinal cord injury arrive immobilized on a spinal backboard with tape/straps from the scene?

- Yes, regularly
- Yes, occasionally
- No, never
- I don't receive patients at high risk of spinal cord injury

1. The time interval(s) for patients with acute spinal cord injury from the scene to your facility includes:

- <6 hours
- 6-12 hours
- 12-24 hours
- 24-48 hours
- >48 hours
- I don't receive patients at high risk of spinal cord injury

1. The cost of diagnostic imaging for spinal trauma at your facility is:

- Free of charge for the patient
- Patients have to pay partially
- Patients have to pay everything
- We don’t have access to spinal imaging services

1. Do you use the ASIA scale for neurologic assessment of acute spinal cord injuries?

- Yes, always
- Yes, occasionally
- No, never

1. For diagnosis, I have available the following options (on site or by an external entity) (more than one option):

- X-ray
- CT regular
- Angio-CT
- MRI
- We don’t have access to spinal imaging services

1. The availability of MRI studies for spinal cord injury cases at my main facility is:

Immediately after the patient’s arrival

- <8 hours from trauma
- 8-24 hours from trauma
- 24-48 hours from trauma
- >48 hours from trauma
- We can’t obtain an MRI
- We don’t receive patients with spinal cord injury

1. Which classification do you use to classify fractures? (more than one option)

- AO-Spine
- SLICS/TLICS
- Both
- None
- Other

1. The costs of spinal surgical procedures at your main facility is:

- Free of charge for the patient
- Patients have to pay partially
- Patients have to pay everything
- We don’t offer spinal surgery services

1. Select the equipment available for spinal surgery in your main facility (more than one option)

- Fluoroscopy
- Surgical microscope
- Surgical loops
- Bipolar and electrocautery
- Skull traction equipment
- Anterior cervical open reduction and fusion equipment
- Open posterior reduction, decompression and fusion equipment
- Occipital-cervical fusion equipment
- Atlanto-axial fusion equipment
- Anterior odontoid screw fixation equipment
- Thoraco-lumbar posterior decompression and arthrodesis equipment (minimally invasive)
- Thoraco-lumbar posterior decompression and arthrodesis equipment (open)
- Thoraco-lumbar anterior/lateral arthrodesis equipment
- Kyphoplasty/vertebroplasty
- Intensive care unit
- All of the above
- Nothing of this equipment list is available at my main facility

1. For the conservative treatment of spinal fractures, in my Institution I have available (more than one option):

- Soft cervical collar
- Hard cervical collar
- Halo-vest
- Braces for thoracolumbar fractures
- Nothing of this device list is available at my main facility

1. Spinal cord injury patients treated in the acute phase (operatively or not), in my main facility are admitted to:

- A general hospital ward
- The neurosurgical ward
- High-dependency unit (HDU)/semi-intensive unit
- Intensive care unit (ICU)
- We don’t receive patients with spinal cord injury

1. Skull traction can be performed at my main facility:

- <8 hours after trauma
- 8-24 hours after trauma
- 24-48 hours after trauma
- >48 hours after trauma
- We don’t have access to skull traction devices
- We don’t use skull traction

1. In our main facility we can operate on patients with spinal cord injury:

- <8 hours after trauma
- 8-24 hours after trauma
- 24-48 hours after trauma
- >48 hours after trauma
- We don’t perform spinal surgery at our main facility
- We don’t receive patients with spinal cord injury

1. If in your facility you have cases of surgical delay (>48h) in spinal cord injury cases, this is related to (more than one option):

- Spinal imaging availability
- Surgical equipment availability
- Transfer times
- We don’t perform spinal surgery at our main facility
- We don’t receive patients with spinal cord injury

1. In my main facility, high-dose corticosteroids are administered to spinal cord injury patients:

- In all cases
- Occasionally, in selected cases (young patients, within 8 hours of injury, 24 hours of infusion)
- Never
- We don't receive patients with spinal cord injury

1. Rehabilitation for spinal cord injury patients is available at my main facility (more than one option):

- During the hospital stay
- After discharge, in dedicated spinal rehabilitation units
- After discharge, in general physiotherapy units
- After discharge, at patient's home
- In my facility/region we don’t have rehabilitation options
- We don't receive patients with spinal cord injury

1. I am aware of the guidelines issued by (more than one option):

- WFNS (World Federation of Neurosurgical Societies)
- DGOU (German Society for Orthopaedics and Trauma)
- CNS/AANS (Congress of Neurological Surgeons/American Association of Neurological Surgeons)
- NICE (National Institute for Health and Care Excellence)
- Other
- None

1. In my daily clinical practice in treating spinal trauma, I feel that I have the possibility to apply the latest recommendations/guidelines/protocols:

- Strongly agree
- Agree
- Neutral
- Disagree
- Strongly disagree

1. If the answer to the previous question was different from strongly agree, rate each option from 0 to 3 (0=no important, 3 very important) the reasons that could limit the application of the latest recommendations/guidelines/protocols. If the answer to the previous question was strongly agree, leave this question in blank.

- Lack/absence of economic resources
- Lack/absence of human resources
- Lack/absence of technology/instruments/equipment
- Lack/absence of infrastructures
- Social/cultural limitations

1. I believe that following the recommendations/guidelines/protocols for spinal trauma can positively affect patients’ outcomes

- Strongly agree
- Agree
- Neutral
- Disagree
- Strongly disagree

1. I believe that flexible, adaptable and resource-targeted recommendations/guidelines/protocol could help me to better treat my patients and, possibly, improving their outcomes

- Strongly agree
- Agree
- Neutral
- Disagree
- Strongly disagree
